# “It Could Become a Standard”—A Qualitative Study of the CARD^TM^ System for Needle-Related Procedures in a Children’s Hospital

**DOI:** 10.3390/children13070951

**Published:** 2026-07-20

**Authors:** Flurina Casaulta, Katrin Marfurt-Russenberger, Adelheid Zeller, Anna Taddio, Janine Vetsch

**Affiliations:** 1 Eastern Switzerland Children’s Hospital, 9000 St. Gallen, Switzerland; 2 Institute of Health Sciences, Eastern Switzerland University of Applied Sciences, 9001 St. Gallen, Switzerland; 3 Leslie Dan Faculty of Pharmacy, University of Toronto, Toronto, ON M5S 3M2, Canada; 4 The Hospital for Sick Children, Toronto, ON M5G 1X8, Canada

**Keywords:** anxiety, CARD, children, education, fear, hospital, needle-related procedures, pain, procedural intervention, self-efficacy

## Abstract

**Highlights:**

**What are the main findings?**

- Using CARD^TM^ in needle-related procedures in a children’s hospital can have multiple positive effects and reinforce the findings from the school vaccination setting.
- CARD^TM^ can support children’s participation in the treatment process and increase self-efficacy.

**What are the implications of the main findings?**

- CARD^TM^ is a promising framework for undertaking needle-related procedures in children’s hospitals.
- This work supports future studies aimed at exploring the impact of using CARD™ in the hospital setting.

**Abstract:**

**Background/Objectives:** Needle-related procedures such as venipuncture are common in pediatric hospitals and are a major cause of pain, fear and distress for those affected. Despite the availability of a wide range of evidence-based support options, it is often the professionals who decide which interventions are offered. In this study, we adapted the CARD^TM^ (Comfort, Ask, Relax, Distract) system for needle-related procedures in hospitals. CARD^TM^ was originally developed to encourage children to actively participate in their vaccination process at school. It allows them to choose from numerous evidence-based interventions in the four different letter categories (C-A-R-D) to reduce pain, fear and related vaccination symptoms. Our aim was to examine perceptions of CARD^TM^ for needle-related procedures in a children’s hospital, including aspects of acceptability, feasibility, self-efficacy in managing fear and pain, and overall experiences. **Methods:** A qualitative descriptive design was used. The sample included nine children aged 10 to 15 undergoing needle-related procedures and seven nurses who performed these procedures. We conducted individual interviews and asked them about their experiences with CARD^TM^. Data were evaluated using qualitative content analysis. **Results:** Four themes were identified: awareness of options and needs; relationship building; empowerment; and acceptability. CARD^TM^ helped children identify available options, prepare themselves, recognize their own needs and select strategies. CARD^TM^ offered a concrete framework for action that promoted participation and self-efficacy and strengthened the relationship between nurses and children. In practice, CARD^TM^ was perceived to be easy to implement and acceptable. **Conclusions:** The present findings suggest the applicability of CARD^TM^ for hospital needle-related procedures.

## 1. Introduction

Needle-related procedures such as blood sampling, venous catheter access or injections are highly prevalent in the clinical routine of pediatric healthcare institutions [1,2,3]. They can lead to numerous short- and long-term negative effects in children, including pre-procedural anxiety, fear, pain, defensive (fight-or-flight) behavior, avoidance of future medical treatment, and increased caregiver stress [1,3,4,5]. In the context of needle procedures, anxiety refers to anticipatory apprehension about a future threat, whereas fear is the emotional response to an immediate perceived threat. Together with pain, these experiences contribute to procedural distress, a broader construct encompassing unpleasant or negative effects during painful or threatening medical procedures [6].

The International Association for the Study of Pain (IASP) defines Pain as “An unpleasant sensory and emotional experience associated with, or resembling that associated with, actual or potential tissue damage” [7]. With the evolution in the understanding of pain—from a simple response to organic tissue damage with intensity proportional to injury to an individually experienced and multifactorial phenomenon [5,8,9]—there is a growing demand for a multidimensional approach to pain management that meets children’s needs [5,9], particularly during long-term treatment or hospitalization [10]. Pain assessment tools, medication guidelines and Non-Pharmacological Interventions (NPIs) that are tailored to the age and developmental stage of each child are promoted by the IASP Special Interest Group on Pain in Childhood (http://childpain.org/. The “Comfort Promise Initiative” includes a fact sheet on bundled interventions for pain management [11]; available at https://www.iasp-pain.org/resources/fact-sheets/pain-in-children-management/ (accessed on 16 June 2026)). In Switzerland, National recommendations are available for NPIs during painful procedures to prevent or reduce pain, anxiety, fear, discomfort and/or distress before, during and after treatment [12].

Current practice in Switzerland suggests that pediatric clinics generally follow these recommendations. Experiences show, however, that it is usually the nursing or medical professionals who choose the coping interventions and there is little opportunity for children to actively participate in the decision-making process [13,14]. Self-efficacy—the ability to cope with new or difficult situations based on one’s own skills [15]—and self-determination may be undermined because children are not informed about the available options, do not have the supportive experience and strategies to successfully cope with the challenging treatments in hospital or are not encouraged to express their needs [14,16,17,18,19]. Children and their parents should ideally receive adequate information about what to expect before needle-related procedures [5,14,17,18,19,20,21]. Preparation can lead to using coping strategies that meet individual needs and preferences, improving the effectiveness of pain management [5,14,17,18,19,20,21]. Interventions are required that give children the opportunity to participate and to use their self-selected coping strategies during the painful procedure [5,14,17,18,19,20,21,22]. Health professionals, in turn, need to be aware of the children’s coping strategies and support their use during the procedure [5,14,17,18,19,21].

As part of a review of the suitability of existing frameworks to encourage preparation and participation of children during needle-related procedures within our hospital, efforts were made to identify tools specifically designed to promote children’s participation and self-efficacy during painful procedures. Through this process, the study team identified the CARD™ (Comfort Ask Relax Distract) system. Key considerations that informed the decision to use CARD™ included: (1) it was co-developed with all knowledge users, including children and caregivers; (2) it is comprehensive, incorporating multiple evidence-based strategies to support children’s coping during needle-related procedures; (3) it is adaptable to individual needs and provides opportunities for preparation and choice for children; and (4) it is supported by evidence from multiple high-quality randomized controlled trials. These characteristics distinguish it from other frameworks. For this project, permission was requested from the lead researcher of CARD^TM^ to translate the tool into German and to adapt it for use in a hospital setting. After the study commenced, a scoping review was identified that also highlighted CARD™ as a promising framework for supporting children during painful procedures [23].

The CARD^TM^ system is a person-centered practice framework that was developed from clinical practice guidelines to help children cope with vaccine injections in the school setting [24,25,26]. Each of the four letters (C-A-R-D) represents a specific category of evidence-based interventions to reduce pain, fear and related symptoms [24,25,26]. It was designed to encourage children to actively participate in their treatment with the knowledge they receive and to choose interventions that are appropriate for them [24,25,26]. Across implementation and effectiveness studies, CARD^TM^ reduced pain, fear and immunization stress-related responses (ISRR), including dizziness and fainting, and improved the vaccination experience for the children and providers at school [24,27,28]. During the COVID-19 pandemic, the CARD^TM^ system was adapted for use in community pharmacies and mass vaccination clinics, where it was similarly demonstrated to be effective and preferrable to standard care approaches. In one of these studies, children aged 5 to 11 years were included, while in the other one children aged 12 years and older and adults were included [29,30].

Due to its simplicity and generally transferable framework with respect to other painful procedures, the creators of CARD™ have recommended expanding it to different procedures and settings [26,31]. This has been examined in a preliminary study conducted in a pediatric dental setting [32]. However, to date, there are no studies exploring the suitability of CARD™ for needle-related procedures carried out in a pediatric hospital setting, in healthcare systems outside North America or in another language. Key process frameworks such as the Knowledge to Action Cycle [33] articulate steps in turning knowledge into action that include evaluating whether innovations are appropriate in different contexts (e.g., setting, population, resources, and culture), and making adaptations to address barriers and facilitators. This study addresses this translational gap by moving from the school vaccination setting for healthy children to the acute care hospital setting, where the context differs substantially in terms of the children’s health status (e.g., acute and chronic medical conditions), the treatment environment (e.g., unfamiliar medical equipment, multiple unknown staff, and higher acuity care), available resources (e.g., nitrous oxide, pain specialists), the complexity of procedures, heightened levels of stress for children and caregivers, and the need for repeated needle-based procedures over an extended period of time that usually cannot be postponed. The aim was to explore children’s and nurses’ perceptions of CARD^TM^ in the context of needle-related procedures conducted within a children’s hospital, including aspects of acceptability, feasibility, self-efficacy in managing fear and pain, and overall experiences.

## 2. Materials and Methods

### 2.1. Material

**Figure 1:**
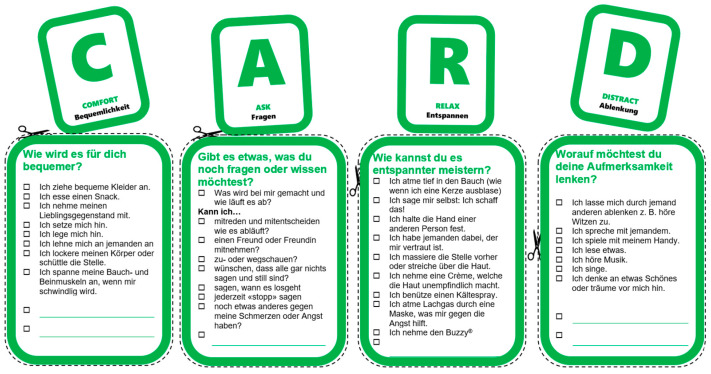
Modified CARD^TM^ pamphlet used for this study (German version).

**Figure 2:**
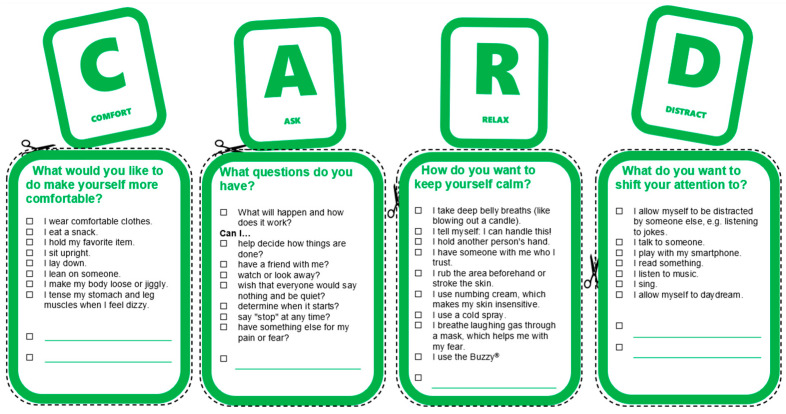
Modified CARD^TM^ pamphlet used for this study (English version).

As part of the development and evaluation of the CARD^TM^ system, the original research team created various educational materials, including a pamphlet for children undergoing vaccinations at school [25,28,34,35,36]. This pamphlet consists of four cards in which the children can select the appropriate interventions for them in advance based on evidence-based suggestions and their own ideas [37]. Then the children “play” their cards during the vaccination [37]. Providers collaborate with the children and support their coping choices [37]. For this study, we adapted the CARD^TM^ selections to include coping options suitable for needle procedures carried out in the hospital setting and translated them into German. Figure 1 and Figure 2 display the CARD^TM^ pamphlet used in the study, in German and English, respectively.

### 2.2. Research Design

A qualitative descriptive design was chosen to answer the research questions. It is the method of choice when a clear description of phenomena is desired [38]. The description is based closely on the participants’ statements and aims to achieve validity of interpretation and agreement from the participants [38]. Reporting followed the Consolidated Criteria for Reporting Qualitative Research (COREQ) (see Appendix A; Table A1).

### 2.3. Sampling and Recruitment

We followed a purposive sampling strategy based on predefined inclusion and exclusion criteria. We included children who were between 10 and 15 years old, had a scheduled outpatient treatment involving a needle-related procedure without narcosis, and had age-appropriate German language skills. We excluded children with severe cognitive developmental disorders, critical health conditions, chronic pain, a first appointment at the outpatient ward, or difficulties understanding German. These criteria ensured that children had prior experiences they could evaluate compared to their experience with CARD™. We separately included nurses who had been trained in the use of CARD^TM^, and who were responsible for caring for the participating children.

We identified eligible children over the recruitment period of three months and asked them and their parents to take part in the study by phone or at clinical appointments. If they agreed, we contacted them by phone or in person and explained the research project to them verbally. They received the written study information letter with the declaration of consent and a copy of the CARD^TM^ pamphlet to fill out in preparation for the upcoming treatment. Before the treatment appointment, we contacted them again to clarify any outstanding questions and scheduled the interview appointment.

We asked the nurses who met the inclusion criteria to participate as soon as consent was obtained from the child. After being informed verbally, the nurses also received the written information letter with the consent form. Then we scheduled their interviews once they had given their consent.

### 2.4. Data Collection

We used the expert interview approach as the interview type to generate information that is representative of the selected group [39]. In this type of interview, the focus is less on the biography of the interviewee and more on their capacity as an expert in a specific field of activity in the respective role [39]. One-on-one semi-structured interviews were conducted by a trained interviewer (Clinical Nurse Specialist graduate student familiar with the topic area and qualitative interviewing) in private (out of range of others) to minimize the influence of onlookers on the perspectives of the participants. Questions probed the perspectives of children and nurses according to their roles (see Appendix B; Table A2 and Table A3). Additional probing questions were used to explore participants’ responses in greater depth. Depending on the preference of the child, a parent could also be present. During the interviews, some parents also shared their thoughts which were part of the data collection. The interviews with the children took place directly after CARD^TM^ had been used in the outpatient ward. The interviews with the nurses took place after the children’s data collection was completed. This meant that we conducted only one interview with the nurses after CARD^TM^ had possibly been used several times with different children, which summarized all experiences. At the beginning of the interviews with children, we collected demographic information, including age and gender. In addition, children self-reported the painful treatment they had come for, their required treatment interval in the outpatient ward and their fear and pain level before and during treatment on a numerical and visual analog scale (VAS range 0–10). We asked nurses who participated about their age, the number of years of professional experience in outpatient care and the total number of times they had used CARD^TM^. All interviews were audio-recorded. During and after the interview, field notes were taken on specific details.

### 2.5. Data Analysis

We conducted a qualitative content analysis. The basic idea behind qualitative content analysis is to maintain the systematic approach to qualitative analysis steps without making hasty quantifications [40]. We transferred the data from the interview recording to the MAXQDA 2020 software and transcribed it. Due to the time lag between data collection in children compared to nurses, we analyzed children’s data first and formed initial categories which were then confirmed and further developed with the data from the nurses. In this sense, data analysis was sequential and cumulative and did not separate the two participant groups. Parents’ comments complemented the children’s accounts and provided additional context during the analysis. We based our approach on the flowchart developed by Kuckartz for systematic data analysis, which consists of seven phases and is described in more detail below with regard to its practical implementation [41]. Following Kuckartz ‘s flowchart we sent the case summaries to three children and three nurses for member checking [41]. All six participants agreed with the content and interpretation (phase 1). To create an initial structure for the data content (phase 2) [41], we derived the main categories a priori deductively from the research questions; however, we added categories inductively, as appropriate, according to the data. Subsequently, we coded all the available data based on the main categories (phase 3 and 4) [41]. For the subcategories, we did an inductive determination and differentiation using coded segments (phase 5) [41]. We ensured that the respective categories were conceptually congruent and distinguishable from other categories. Sub- and main categories were partially dissolved, summarized, renamed and redefined or assigned to a different category (phase 6) [41]. In addition, we compared the coding of one transcript by ourselves and by an external coder to check the consistency of the assignment of coded text passages and to improve accuracy and selectivity. In the last step (phase 7), we analyzed the data based on the main categories [41]. For this purpose, we read all text segments of the respective subcategory and systematically summarized them in terms of content. Overall, categories were developed using a deductive–inductive approach, and trustworthiness was ensured through multiple strategies including member checking and independent coding.

### 2.6. Ethical Considerations

The study was reviewed by the Institutional Ethics Committee of Eastern Switzerland (EKOS) and participation was voluntary. Following the guidelines of swissethics [42], we informed the 10-year-old children about the research project verbally and the parents received additional written information for consent. The children aged 11 to 13 also received written, age-appropriate information [42]. The parents received the complete information for consent [42]. Children aged 14 and older also received the written information [42]. They were able to consent to participate in the study independently and without parental consent, as we considered the potential for risk and stress low [42]. To minimize stress, we conducted the CARD^TM^ intervention whenever possible through the children or trusted nurse and in the presence of a parent, depending on the children’s habits. The children received a movie voucher in recognition of their participation in the study.

The participating nurses gave their consent after receiving verbal and written information. The nurses were compensated for the time required to participate in the study as working hours.

### 2.7. Generative Artificial Intelligence

No generative artificial intelligence (GenAI) was used in this study (e.g., to generate text, data or graphics, or to assist with study design, data collection, analysis or interpretation).

## 3. Results

### 3.1. Description of Participating Children and Adolecents

We asked fourteen children to participate between November 2021 and January 2022 and nine children (five males) agreed. Two children initially consented, but revoked consent because they had too much schoolwork, and three children refused to participate. The participating children were on average twelve years old with an age range of 10 to 15 years. They were admitted for needle-related procedures such as venous blood sampling (five) or venous catheter access (four). All procedures were successfully completed on the first attempt. The average required treatment interval in the outpatient clinic was around every two months. Except for one child, all the others had a parent present during the treatment appointment and interviews. The interviews lasted a median of 26 min, ranging from 11 to 39 min. For infusion treatments lasting several hours, the interviews took place directly in the treatment room during the ongoing therapy or, in the case of venous blood samples, immediately after the treatment in a single room.

### 3.2. Description of Participating Nurses

All seven nurses involved agreed to participate. Two of them had used CARD^TM^ more than once. The median age was 44 years, the median years of general work experience was 20 years, and they had been working in the outpatient ward for 9 years. The interviews took place during regular working hours at off-peak hours or on office days. The median interview duration was 44 min, with a range between 19 and 57 min.

### 3.3. Description of Themes, Subthemes Based on the Research Questions

The experiences of children and nurses using CARD™ were categorized in four themes with nine associated subthemes (summarized in Table 1 with selected related quotes). The themes included: awareness of options and needs; relationship building; empowerment; and acceptability. The summary of the content follows after the table.

### 3.4. Theme 1: Awareness of Options and Needs

#### 3.4.1. Overview of Available Options

Both children and nurses reported finding CARD™ useful for demonstrating ways to make needle-related procedures as comfortable as possible. They remarked that the listed suggestions and associated knowledge transfer are particularly helpful for inexperienced children. Children viewed CARD^TM^ as a tool for learning about something new or different, such as choosing their clothes more purposefully or being accompanied by a friend. Furthermore, nurses commented on its ability to stimulate children to think about their own coping ideas.

#### 3.4.2. Preparation and Strategy—What Is Important and Necessary?

Participants reported that CARD^TM^ gave children the opportunity to prepare and recognize what is important to them for their treatment. Children were encouraged to think about their own needs as well as gain clarity about the treatment process. Being aware of their options from the outset means children did not have to make decisions “on the fly” and when under pressure or experiencing distress. The nurses stated that the preparation enabled children to identify possible coping strategies for themselves, which they could then apply in the challenging situation of treatment. In addition, this allows children to recognize their own resourcefulness.

### 3.5. Theme 2: Relationship Building

#### 3.5.1. Being Heard and Encouraged to Participate Actively

Using CARD™ created opportunities for time, communication, and relational engagement with children. The nurses found it helpful to sit down with the child and discuss CARD^TM^, which allowed for more meaningful interaction than when briefly explaining the procedure. They expressed a stronger sense of cooperation. One nurse stated that she noticed an increased willingness on the part of the child to engage in the interaction. Another nurse described CARD^TM^ as an ‘icebreaker’ (Nurse 01). CARD^TM^ enabled them to connect with children through structured questions and gain insight into each child’s preferences. These conversations helped nurses better understand the child and support rapport and trust during the procedure.

The nurses felt that CARD^TM^ made children (and their parents) feel that their needs and preferences were being taken more seriously. They reported that someone was actively asking about their needs, listening and taking time for them so that they could cope with the challenge. The nurses also said that CARD^TM^ encourages children to actively participate in shaping the treatment process and to express their needs and wishes.

#### 3.5.2. Feeling Secure Through Common Agreement

With CARD^TM^, nurses and children had a tangible tool to guide communication and structure for the treatment process. CARD™ helped clarify children’s options and allowed nurses to implement selected strategies step by step following a brief discussion or confirmation before the procedure. This reduced the need to consider alternative approaches in the moment and enabled nurses to focus on the child’s needs. According to the participants, the children felt more secure and in control, trusting that the process was organized according to their needs. Nurses felt confident because they knew that they were supporting children in accordance with their chosen strategies and were not offering them anything that was not suitable for them. The nurses no longer had to present their entire repertoire of strategies but could focus on responding to the children’s needs. Children could also spontaneously change their decisions during the procedure without disrupting the process.

### 3.6. Theme 3: Empowerment

#### 3.6.1. Influence on Child’s Fear and Pain Level

Most children stated that, due to their extensive prior experience with needle procedures, they no longer had a significant fear of the procedure. Many said that they had been more fearful when they were younger or during their first hospital appointments. Some reported that they still felt fear in the presence of unfamiliar professionals or when they could not remember their last procedure. They agreed that even if they themselves did not experience a reduction in fear, CARD^TM^ could be beneficial for other children experiencing fear. Nurses believed that even children who did not express fear were more relaxed during treatment because they were actively involved. Two children, who rated their pre-procedural anxiety as 8 and 10 on the VAS, described fear before using CARD^TM^. In one child, this manifested as a strong feeling of insecurity and discomfort when thinking about the injections. The other child expressed fear in the form of intense panic and resistance to treatment, which they could only endure with a sedative that left them very sleepy for the rest of the day. Following implementation of CARD™, both children described feeling calmer and better able to cope with the procedure, reporting that their fears did not escalate to the same extent as in previous experiences. They attributed these changes to preparing with CARD™ and to the nurse following their selected coping strategies during the procedure. Similarly, the two nurses perceived these children to exhibit less procedural distress. These accounts illustrate how individualized preparation and participation in decision-making may enhance children’s sense of control and coping during needle procedures.

Similarly to their experiences with fear, most children reported experiencing little or no pain during the treatment. Although they reported feeling the needle penetrate the skin, they did not associate this with pain. The same two fearful children experienced moderate to severe pain during treatment and rated the pain as 5 and 7, respectively, on the VAS. Following discussion of CARD™ with the nurse, the child with a pain rating of 7 elected to be pretreated with a topical anesthetic (EMLA^®^ patch); this child believed that the patch, together with their supine position, helped to alleviate the pain. The other child used a self-affirmation strategy involving positive thoughts instead of medication and perceived this approach to reduce the pain experienced during the procedure; the attending nurse shared this impression. These individual accounts illustrate how CARD™ may support children in identifying and using coping strategies that they perceive as helpful, rather than demonstrating the effectiveness of any specific intervention.

#### 3.6.2. Self-Efficacy—Coping with Challenging Situations

Most participating children expressed a high level of self-efficacy upon arrival at the hospital, regardless of their current use of CARD^TM^, as they were familiar with hospital care. They knew what to expect, had found suitable strategies for their needs, and were able to advocate for themselves. This view was confirmed by the nurses.

As mentioned above, two of the children (Children 06 and 07) expressed concerns before they were introduced to CARD^TM^. This happened when they realized they had to go to hospital. One child felt uncomfortable as soon as the hospital came into view. The other child expressed panic and wanted to refuse treatment. With CARD^TM^, both children were able to successfully undertake the procedure with increased confidence and self-efficacy. The nurses mentioned that CARD^TM^ probably could not dispel all concerns, but that the child would learn to deal with their fear and say what they specifically needed. According to the participants, CARD^TM^ enabled them to take more responsibility for their own decisions. The children learned to speak up for themselves and put their feelings into words, regardless of whether their parents were present.

The children stated that young and shy children tend not to express their needs or dare to intervene when something is not right for them. They felt that CARD^TM^ helped them to participate more actively in decision-making and to speak up when they need something, rather than just agreeing (i.e., to advocate for themselves).

### 3.7. Theme 4: Acceptability

#### 3.7.1. Necessary Conditions and Feasibility

The nurses mentioned that using CARD^TM^ added time to discussing the treatment process with the child, which was considered feasible. In addition, children required a certain level of reading comprehension because the pamphlet that was evaluated used words to educate about CARD™. Nurses felt that children should be receptive to CARD^TM^ before treatment, despite their heightened emotional state, and that its use should not increase fear. The implementation of CARD^TM^ did not pose any difficulties for nurses, as they were already familiar with listed interventions, had used them before, and found them easy to integrate into daily practice.

The children considered CARD^TM^ suitable for children of the same age, but also for younger children who may be more fearful. The participants primarily viewed CARD^TM^ as a selective tool to help children who need more support and for whom the usual approach is not sufficient, such as children with pronounced fear or children who are too shy to express their own needs. They also believed that children and their parents could benefit from CARD^TM^ when they first come to the hospital and thereafter, when they have little experience with painful procedures.

#### 3.7.2. Comprehensibility

The nurses found CARD™ easy to understand and user-friendly. They stated that the children quickly grasped the principle of selecting from the acronym without additional explanations. Most children were able to complete their coping selections independently and understood the content, meaning and purpose. In some cases, they were assisted by a parent to clarify individual points. For all study participants, the proposed interventions reflected an appropriate selection of interventions that can be offered to children in hospital during painful treatments. There were some suggestions for additions or changes to the interventions on CARD^TM^, such as adding a ‘balancing bird’ for distraction, a plaster solution spray, or the option to write down a reward ritual after mastering the treatment.

The four existing C-A-R-D strategy areas were considered useful. The ability to cut them out was seen as an additional ‘fun factor’. Overall, participants had mixed opinions about the layout design. Some found the text-heavy design difficult to read. Others appreciated the clarity and structure. They suggested that each individual CARD^TM^ should have a different color.

#### 3.7.3. Perceived Benefit

For the nurses, CARD^TM^ is a way to improve the quality of care and well-being of the children. Based on the positive feedback by children and parents, they also found that it offered concrete benefits and helpful support, including for themselves. They believed that CARD^TM^ could become established as the standard of care in the long term.

The children enjoyed using CARD^TM^. Together with their parents, they were convinced of its positive effects. Only one child was not motivated to use CARD^TM^ and did not see a benefit. Based on their experiences, all participating nurses would recommend CARD^TM^ to other nurses who care for children during painful procedures. They would also like to continue using CARD^TM^ themselves to gain further experience. With one exception, all participating children stated that they would recommend CARD^TM^ to their friends if they had to go to the hospital for painful treatments.

## 4. Discussion

The results of this qualitative study provide valuable insights into the potential applicability of the CARD^TM^ system for needle-related procedures in a children’s hospital. Participants found CARD^TM^ helpful in showing children what support is available, preparing them for these procedures, recognizing their own needs and determining the strategies that are appropriate for them. CARD^TM^ offered them something concrete that focused on the children’s individual needs and provided them with guidance during the treatment process. Supported by the necessary time investment and active involvement, there could be the potential to strengthen the relationship between nursing staff and children as well as to increase children’s active participation. For children with existing fears and pain, there were perceptions that CARD^TM^ helped improve their well-being and increase their self-efficacy. In practice, CARD^TM^ proved to be easy to implement and understand, with temporal and technical feasibility but requires the willingness of children to actively address their needs and fears they may have. As a consequence, the positive experiences with CARD^TM^ led to a high level of acceptance among participants in terms of willingness to recommend it to peers.

The current results are consistent with previous qualitative and quantitative studies with CARD ^TM^. With regard to the theme of **‘Awareness of options and needs’**, previous studies demonstrated a similar benefit on preparedness [24,28,31,34,43]. Children not only knew which coping strategies they preferred, but were additionally able to change their minds spontaneously during the procedure [28,31,43,44]. With CARD^TM^, the children were also given more coping options by the treatment team [43,45]. Appropriate preparation, including teaching coping strategies, encouraging children to face an upcoming procedure [1], and aiming to convey realistic expectations about a procedure can build trust, provide a sense of control, and promote self-efficacy in coping [46]. In future, it will be important to consider the right time for preparation with CARD^TM^. For school-age children, who are typically afraid of needles, this is likely ahead of time, when the child is not yet feeling pressure or stress about the upcoming procedure. Studies show that even short-term education in coping strategies, for example, in emergency situations, can be helpful [20]; others recommend preparation a few days in advance [1]. One advantage of preparing ahead of time is the opportunity to wear comfortable clothing and bring aids such as favorite comfort and distraction items from home.

**‘Relationship building’** was identified as key to successful collaboration between nurses and families. These results are consistent with previous CARD™ studies, which reported a strengthened relationship based on interest, active listening and fostering trust by addressing participants’ concerns [32,34]. In addition, nurses reported improved collaboration with children and felt that they were more actively involved in their treatment with CARD™ [24,45]. The nurses themselves were able to focus on patient-centered care through coaching during the procedure [24,45]. CARD™ helped nurses better understand children’s needs and was described as a way to give them a voice, especially shy children [43]. Nurses reported more pleasant interactions with the children [24,31], and children were able to advocate for their own preferences rather than simply following instructions [31,32,43]. The opportunity to verbalize their needs and be included in the decision-making process gave them a greater sense of control and personal responsibility, which improved their overall experience during vaccination [32,44].

Nevertheless, it is important to point out that some nurses in prior CARD™ studies reported that they used the usual coaching approaches for vaccinations, instructing the children rather than engaging them in making their own choices [45,47]. They expressed skepticism about the idea that children should take the lead with their own coping strategies, especially if they were not convinced by the chosen intervention [47]. Some children stated that nurses did not always take a patient-centered approach and did not respond to their needs [28,43]. Although there was no evidence of such attitudes in this study, the attitude of nurses is certainly something that should be considered in future implementation efforts. It should be noted that children cannot experience self-efficacy without active participation in the treatment process. Furthermore, a patient- and family-centered approach is regarded a fundamental characteristic and component of pediatric care [48], as is taking into account the rights of sick children as described in the Charter of the European Association for Children in Hospital [EACH] Charter (https://each-for-sick-children.org/each-charter/, accessed on 17 September 2021) [49].

This study found preliminary evidence that CARD™ could have a positive impact on fear and pain perception and increase participants’ self-efficacy, as summarized in the theme ‘**Empowerment**’. Results from earlier studies showed positive and mixed results for fear, pain and other stress-related responses (e.g., dizziness) from CARD™ [30,36,47]. Discrepant results may be explained by differences in sample size, vaccine recipient demographics, as well as vaccine setting. Qualitative statements, however, are mostly positive [24,28,31,45]. Nurses described children as calmer [24,29,31], and believed that it was an important benefit to talk openly about fear and to normalize it [24,31,43,47], as well as teach skills for dealing with challenging situations [28,32,34]. These findings underline the relevance of addressing both fear and pain in children undergoing needle-related procedures due to their inter-relational nature, with fear often greater than pain [50]. Prior CARD™ studies also demonstrate that nurses who used CARD™ reported greater self-efficacy. This was associated with greater confidence in their ability to recognize and manage children’s pain and fear, and the knowledge that the children’s needs were being met [24,29,31].

In terms of the theme **‘Acceptability’**, previous studies have shown that CARD™ is generally well accepted by users and confirm many aspects of the present study. CARD™ was reported as easy to understand, with just the right amount of information and appropriateness, clearly presented, easy to use, and liked [24,28,31,36,43,45]. As in the current study, nurses reported that CARD™ builds on their practical experience and knowledge, and reflects this in a validated and formalized manner [24,29,31,45]. Due to the positive experiences associated with CARD^TM^, acceptance in terms of willingness to use and to recommend to peers was generally high, and the participants wanted to continue using CARD™ in practice [24,31,36,43]. Recommendations have been made about altering formats for learning about CARD™ for use in different contexts, including using checkboxes with options for older children, illustrations for younger children, the use of a digital platform, the inclusion of interactive activities, and the creation of a pocket-sized card [24,31]. It should be noted that since this study was conducted, CARD™ has been updated with respect to the range of application materials and teaching methods. Most of them are posted at www.cardsystem.ca and available in various languages. This includes visual CARD™ coping checklist pamphlets, a CARD™ web game for children to introduce them to CARD™ and support pre-procedural preparation or distraction during the procedure, as well as a CARD™ e-learning module for providers.

In the present study, only one child did not perceive CARD™ as beneficial, which may be explained by a low level of fear of needles and extensive experience with needle procedures that shaped their perceived need for the intervention. Some nurses in previous studies have similarly expressed uncertainty regarding the role of CARD™ for children who are not fearful, suggesting variability in perceived relevance depending on patient characteristics [43,44,45,47]. In this context, it is important to question the starting point for application—whether CARD™ should be used as a general framework for all children or applied selectively for those with higher levels of need or distress. CARD™ is broadly applicable as a healthcare delivery framework, as it is inherently based on an individualized approach to care. Moreover, the fact that a child does not actively select specific coping strategies does not imply that being asked about preferences is irrelevant; rather, the process of eliciting preferences may itself be a meaningful component of care and support. Many healthcare professionals may not yet be aware that NPIs must be personalized to be effective [51]. If healthcare professionals selectively decide who is suitable for CARD^TM^, this is akin to a paternalistic approach in which decisions are made without involving the child. It was also recognized that CARD™ was helpful not only for children who were visibly anxious, but also for others who did not initially appear anxious [29]. Fear manifests in various ways and is not always immediately apparent to carers. It is certainly essential that all children and their families should be informed about CARD^TM^ as a standard and can decide for themselves in which situations they want to use it, knowing that the nurses are also aware and will provide appropriate support.

This study has several strengths and limitations. The relevance of the topic in pediatrics is great due to its clinical frequency. With two respondent groups with experiences with hospital-based needle procedures totaling 16 participants, a certain degree of data saturation was achieved in the analysis within the scope of the sampling, with signs of repetition in terms of content. Trustworthiness was enhanced through the use of a structured qualitative content analysis approach, supported by member checking, iterative category refinement, and independent coding of a transcript to ensure consistency. While the results provide initial support for the applicability of CARD™ in the hospital setting, it should be noted that the sample was selected specifically, and the results are of limited transferability to other contexts. Most participating children had learned how to successfully cope with the challenges of painful procedures during their illness. This had the advantage that their expertise could be incorporated into the evaluation of the applicability of CARD^TM^ but was generally disadvantageous for proving the effectiveness of CARD^TM^, as the children already had a deep understanding of coping with painful needle procedures. Their statements on these aspects were therefore often hypothetical in nature.

## 5. Conclusions

This study, undertaken in a pediatric hospital setting, demonstrated that children and nurses had positive perceptions about using CARD™ during needle procedures. This work supports future studies aimed at exploring the impact of more fulsome and systematic integration of CARD™ in the hospital setting, including clinical and implementation outcomes (e.g., pain, fear, satisfaction, feasibility, acceptability, and fidelity) among children, caregivers and nurses.

## Figures and Tables

**Table 1:** Summary of themes and related subthemes with related quotes.

Themes	Subthemes with Related Quotes
**Awareness of options and needs**	**Overview of available options** *“I hadn’t even thought about bringing a friend along.” (Child 02)* *“* *… or that they [inexperienced children] know what they can do. When they come here for the first time, they don’t know what they can do. They don’t know whether they are allowed to play with their cell phones or not. With CARD^TM^, they just know.” (Child 09)* **Preparation and strategy** **—** **what is important and necessary?** *“CARD^TM^ are very good because she reads it again, she can think about it, ok this is how it is, that fits. And that’s why it’s very good for her.” (Father Child 06)* *“And they can prepare well, they can think in advance, in a calm situation, and see what they need when it comes to an unpleasant intervention that triggers stress and anxiety.” (Nurse 01)*
2. **Relationship building**	**Being heard and encouraged to participate actively** *“* *… yes, simply with the idea that you are being asked or it is also an invitation to think and say something. I think that’s the beauty of it. When you don’t necessarily know that you’re allowed or able to do that.” (Mother Child 05)* *“And funnily enough, one of the patients came back a week or two later. She wasn’t even in my office, but she recognized me from afar and waved at me. On other occasions, you probably wouldn’t have experienced such encounters or such a relationship developing in such a short time.” (Nurse 04)* **Feeling secure through common agreement** *“* *… it [the child] could tick boxes on the CARD^TM^ and you could respond to these points and also implement them 1:1, (…). You didn’t have to think long about whether another solution might have been better or something like that (…) it also takes the pressure off me somehow, because I don’t have to think myself what would be good for the child and in the end, I decide on a wrong option that makes no sense for the child at all.” (Nurse 06)*
3. **Empowerment**	**Influence on child’s fear and pain level** *“When I really get into it, it’s already at ten, but today it was at two to zero. So in between.” (Child 06)* *“Well, I don’t know now whether these CARDs^TM^, if you can choose what you want, whether they help against the pain, but if you know what you have to do to make it not hurt, then yes.” (Child 08)* **Self-efficacy** **—** **coping with challenging situations** *“She [the child] dealt with it [CARD^TM^] and then she also realized that it did help her. And it was a huge difference compared to before. Before she had it, and from the moment she was able to start working with it [CARD^TM^]” (Nurse 03)* *“Yes, I think she has also learned to express herself. In other words, to be able to verbalize her feelings. I think she also learned that a bit through CARD^TM^.” (Nurse 03)*
4. **Acceptability**	**Necessary conditions and feasibility** *“CARD^TM^ are helpful if you use them properly and take them seriously, yes.” (Child 09)* *“And I mean these are all things that are in our repertoire, zero problem to implement. So, I think (…) It’s actually nothing new for us.” (Nurse 03)* **Comprehensibility** *“Well, I did it with my mom, […] but I could have done it on my own.” (Child 01)* *“I associate green with hospitals because there’s a thing with a green snake in front of the pharmacy. I would like each card to be a different color.” (Child 08)* **Perceived benefit** *“I would let her [a friend] read through it and say: “Yes, you can use that” or that’s so and so. I would give it to her. Because I found it good and useful myself.” (Child 07)* *“* *… there are sometimes things that fizzle out, but I can already imagine that this [CARD^TM^] (…) it could be a standard in terms of handling.” (Nurse 04)*

## Data Availability

The raw data supporting the conclusions of this article will be made available by the authors on request due to privacy.

## Appendix A

**Table A1:** Consolidated Criteria for Reporting Qualitative Research (COREQ) checklist [52].

Item No. and Topic	Guide Questions/Description	Response and Reported on Page No.
Domain 1: Research team and reflexivity		
*Personal characteristics*		
1. Interviewer/facilitator	Which author conducted the interviews?	Casaulta, F.
2. Credentials	What were the researcher’s credentials? e.g., PhD, MD	Casaulta, F.: MSc Marfurt-Russenberger, K.: MSc Taddio, A.: PhD Vetsch, J.: PhD Zeller, A: PhD
3. Occupation	What was their occupation at the time of the study?	Casaulta, F.: Clinical Nurse Specialist, Eastern Switzerland Children’s Hospital Marfurt-Russenberger, K.: Head of Nursing Quality and Development, Eastern Switzerland Children’s Hospital Taddio, A.: Professor, Leslie Dan Faculty of Pharmacy, University of Toronto; Senior Associate Scientist, The Hospital for Sick Children, Toronto, Vetsch, J.: Head of the Competence Centre for Evidence-Based Healthcare, Eastern Switzerland University of Applied Sciences Zeller, A: Professor, Competence Centre for Dementia, Eastern Switzerland University of Applied Sciences
4. Gender	Was the researcher male or female?	Female
5. Experience and training	What experience or training did the researcher have?	The interviewer had prior experience in qualitative interviewing and pediatric nursing.
*Relationship with participants*		
6. Relationship established	Was a relationship established prior to study commencement?	No
7. Participant knowledge of the interviewer	What did the participants know about the researcher? e.g., personal goals, reasons for doing the research	The participants and parents of the participating children were informed about the reasons for conducting the research through verbal explanations and a written information letter.
8. Interviewer characteristics	What characteristics were reported about the interviewer/facilitator? e.g., Bias, assumptions, reasons and interests in the research topic	Name, professional role
**Domain 2: Study design**		
*Theoretical framework*		
9. Methodological orientation and Theory	What methodological orientation was stated to underpin the study? e.g., grounded theory, discourse analysis, ethnography, phenomenology, content analysis	Qualitative research design/page 4
*Participant selection*		
10. Sampling	How were participants selected? e.g., purposive, convenience, consecutive, snowball	Purposive sampling/page 5
11. Method of approach	How were participants approached? e.g., face-to-face, telephone, mail, email	Face-to-face, telephone/page 5
12. Sample size	How many participants were in the study?	9 children, 7 nurses/page 7
13. Non-participation	How many people refused to participate or dropped out? Reasons?	5 children, too busy at school or not in the mood/page 7
*Setting*		
14. Setting of data collection	Where was the data collected? e.g., home, clinic, workplace	rooms within the children’s hospital/page 5
15. Presence of non-participants	Was anyone else present besides the participants and researchers?	One parent for each of 8 participating children/page 7
16. Description of sample	What are the important characteristics of the sample? e.g., demographic data, date	Children: Age, gender, treatment procedure, interval of treatment Nurses: Age, gender, general work experience and practice in the current research setting/page 5
*Data collection*		
17. Interview guide	Were questions, prompts, guides provided by the authors? Was it pilot tested?	Semi-structured interview guide/page 5. Interview guides provided/Appendix B
18. Repeat interviews	Were repeat interviews carried out? If yes, how many?	No
19. Audio/visual recording	Did the research use audio or visual recording to collect the data?	Audio-recorded/page 5
20. Field notes	Were field notes made during and/or after the interview?	Yes. During and after the interviews/page 5
21. Duration	What was the duration of the interviews?	Children: median = 26 min/page 7 Nurses: median = 44 min/page 7
22. Data saturation	Was data saturation discussed?	Yes/page 14
23. Transcripts returned	Were transcripts returned to participants for comment and/or correction?	Yes. Three children and three nurses each received their case summaries for the member check/page 6
**Domain 3: Analysis and findings**		
*Data analysis*		
24. Number of data coders	How many data coders coded the data?	2 and 1 external coder/page 6
25. Description of the coding tree	Did authors provide a description of the coding tree	Yes, upon request/page 14
26. Derivation of themes	Were themes identified in advance or derived from the data?	The themes were determined in advance a priori deductively from the research questions, as well as empirically inductively from the data/page 6
27. Software	What software, if applicable, was used to manage the data?	MAXQDA 2020 Software/page 6
28. Participant checking	Did participants provide feedback on the findings	No
*Reporting*		
29. Quotations presented	Were participant quotations presented to illustrate the themes/findings? Was each quotation identified? e.g., participant number	Yes, presented in Table 1/page 7–8
30. Data and findings consistent	Was there consistency between the data presented and the findings?	Yes, presents in discussion
31. Clarity of major themes	Were major themes clearly presented in the findings?	Yes, presented in results
32. Clarity of minor themes	Is there a description of diverse cases or discussion of minor themes?	Yes, presented in discussion

Developed from: ref. [52].

## Appendix B

### Appendix B.1

**Table A2:** Interview guide Children (translated from German).

Main Questions	Sub-Questions/Probes
What did you think when you saw CARD^TM^ for the first time?	What was going through your mind?
How did it go for you today when you used CARD^TM^ for your treatment?	What was helpful? OR What did not help?
If you have to go to the hospital with your parents for a procedure involving a needle, how does that make you feel?	How confident are you in your ability to cope during the needle? Can you explain your answer. What do you do to cope during the needle?
Did you notice any changes with CARD™?	Does CARD^TM^ help you undergo a procedure? Can you explain your answer. Why yes? Why not?
How scared did you feel during the treatment today?	On a scale of 0 to 10, how would you rate your fear? (Show VAS) Can you explain why you were afraid/not afraid?
How did CARD^TM^ affect your fear today?	Describe any changes from treatments you have had before.
How much pain did you feel during the treatment today?	Can you also rate your pain during the treatment on a scale of 0 to 10? (Show VAS) Can you explain why you had/did not have pain?
How did CARD^TM^ affect your pain today?	Describe any changes from treatments you have had before.
What effect, if any, did CARD™ have on how you prepared for and coped with your treatment today?	What differences did you notice compared to treatments without CARD^TM^? How well prepared did you feel for the procedure with CARD™? Can you explain your answer.
Were there times that you got to make a choice or share what you wanted during the treatment today?	Can you explain your answer.
How important is it for you to have a say in how your treatment is carried out?	Do you think CARD™ made any difference to making choices or telling the nurse what you wanted? Why or why not?
How helpful was CARD^TM^ to you during your treatment?	Can you explain your answer.
How easy was it for you to use CARD^TM^ ?	Did you fill out CARD^TM^ on your own? What did you need help with? Was anything hard to understand?
How helpful were the coping interventions described under each letter category?	How do these coping interventions match what you did? What would you add or change to how CARD™ is presented (size, labeling, other)?
Is there anything you would like to change about CARD^TM^?	What would that be, specifically?
How should we use CARD^TM^ in the hospital?	In your opinion, which children could benefit from CARD^TM^? In your opinion, which children don’t need CARD^TM^ or would have difficulty using it?
Would you recommend CARD^TM^ to your friends if they had to go to the hospital for procedures involving needles?	Can you explain your answer. Why yes? Why not?
Would you like to continue using CARD^TM^ if you ever need another treatment with a needle?	Can you explain your answer. Why yes? Why not?
Is there anything else you’d like to tell me about CARD^TM^ that I haven’t asked you about yet?	

### Appendix B.2

**Table A3:** Interview guide Nurses (translated from German).

Main Questions	Sub-Questions/Probes
What did you think when you saw CARD^TM^ for the first time?	What was going through your mind?
What was it like for you when you used CARD^TM^ with the children for their treatment?	What changes did you notice when using CARD^TM^ compared to before using it? What was good about using CARD™? What was not good about using CARD™?
What do you think is challenging for children when they come to the hospital for needle-related procedures?	How do you perceive/notice these challenges? How do you handle/deal with these challenges?
What role does CARD^TM^ play for you in supporting children successfully overcome these challenges?	What changes did you notice with CARD^TM^? Can you explain your answer.
How afraid do you think children were about their treatment?	How did the children show it? Why do you think the children were/were not afraid?
What do you think was the impact of CARD^TM^ on their fear?	How, if at all, do you think using CARD™ affects fear? Can you explain your answer.
How much pain do you think children experienced from the treatment?	How did they show it? Why do you think they were/were not in pain?
What do you think was the impact of CARD^TM^ on their pain?	How, if at all, do you think using CARD™ affects pain? Can you explain your answer.
How prepared were children after using CARD^TM^?	How does this differ compared to treatments without CARD^TM^? Can you explain your answer.
How important is it for you to have children actively participate in their procedure?	How does it make you feel when children are involved in their treatment? What role did CARD^TM^ play in this? How did/does this compare to treatments without CARD™?
How useful is CARD^TM^ to you as a nurse?	Can you explain your answer.
How easy was it for you to use CARD^TM^?	Can you explain your answer.
How easily can CARD™ be incorporated into routine practice?	How much help did the children need to understand and use CARD™? What did you have to do to help? What else is needed to be able to use CARD^TM^ in routine practice?
How helpful do you find the interventions described in CARD™?	How well do these interventions match your experience and practice? What would you add or change (size, labeling, other)?
Is there anything you’d like to change about CARD^TM^?	Can you explain your answer?
How should we use CARD^TM^ in the hospital?	Which children could benefit from CARD^TM^? Which children do not need CARD^TM^ or would have difficulty using it?
In your opinion, what are the reasons why nurses should or should not use CARD^TM^?	What are the benefits/pros? What are the drawbacks/cons?
Would you recommend CARD^TM^ to other nurses who care for children who must undergo needle-related procedures?	Can you explain your answer. Why yes? Why not?
Would you like to continue using CARD^TM^ for needle procedures?	Can you explain your answer. Why yes? Why not?
Is there anything else you’d like to tell me about CARD^TM^ that I haven’t asked you about yet?	
